# Investigation into the Use of Surufatinib and Donafenib as Novel Multi-Kinase Inhibitors Therapeutic Agents in Managing Advanced Differentiated Thyroid Cancer: A Systematic Review

**DOI:** 10.3390/biomedicines13030752

**Published:** 2025-03-20

**Authors:** Danut Dejeu, Paula Dejeu, Anita Muresan, Paula Bradea, Viorel Dejeu

**Affiliations:** 1 Surgical Oncology Department, Emergency County Hospital Oradea, Strada Gheorghe Doja 65, 410169 Oradea, Romania; ddejeu@uoradea.ro (D.D.); amuresan@uoradea.ro (A.M.); 2 Surgery Department, Faculty of Medicine and Pharmacy, University of Oradea, Piata 1 Decembrie 10, 410073 Oradea, Romania; 3 Bariatric Surgery Department, Medlife Humanitas Hospital, Strada Frunzisului 75, 400664 Cluj Napoca, Romania; 4 Laboratory Medicine Unit, Betania Medical Center, Menumorut 12, 410004 Oradea, Romania; 5 Gastroenterology Unit, Betania Medical Center, Menumorut 12, 410004 Oradea, Romania; paula.bradea@betania-centrulmedical.ro; 6 Bariatric Surgery Department, Life Memorial Hospital, Calea Grivitei 365, 010719 Bucuresti, Romania; office@doctordejeu.ro

**Keywords:** surufatinib, donafenib, multi-kinase inhibitors, systematic review, progression-free survival, objective response rate

## Abstract

**Background and Objectives:** Differentiated thyroid cancer is the predominant form of endocrine cancer, with most cases being treatable. However, some patients develop resistance to traditional treatments. This review examines the use of the new multi-kinase inhibitors surufatinib and sonafenib, which target pathways related to angiogenesis and tumor growth in these patients. **Methods:** An extensive search of the literature was performed to find research involving these drugs in treating differentiated thyroid cancer. Four relevant studies were found, including two each for surufatinib and donafenib. Information regarding the research design, participant details, treatment methods, results on effectiveness, and side effects was collected and analyzed. **Results:** Surufatinib showed encouraging results, with response rates between 23.2% and 60% and progression-free survival times as long as 11.1 months. Donafenib also demonstrated improved progression-free survival times (12.9 months) compared to a placebo (6.4 months) and had response rates as high as 23.3%. Both drugs were well tolerated, with the most frequent side effects being hypertension and hand−foot syndrome. **Conclusions:** Both urufatinib and donafenib offer substantial benefits for patients with advanced differentiated thyroid cancer and have acceptable safety profiles. These results support their potential inclusion in treatment strategies for resistant cases, and further investigation of their clinical application is recommended.

## 1. Introduction

Differentiated thyroid cancer (DTC) encompasses papillary and follicular thyroid carcinomas and represents the most common type of thyroid malignancy, accounting for over 90% of cases [1]. The incidence of thyroid cancer has been increasing globally, with significant increases observed in China, which accounts for approximately one-third of new cases worldwide [2,3]. While the prognosis for DTC is generally favorable, with a 5-year survival rate exceeding 90%, a subset of patients develop advanced disease that is refractory to conventional therapies, such as surgery, radioactive iodine (RAI) therapy, and thyroid-stimulating hormone (TSH) suppression [4,5].

Radioactive iodine-refractory differentiated thyroid cancer (RAIR-DTC) poses a significant clinical challenge [6]. Patients with RAIR-DTC have limited treatment options and poor prognosis, with a 10-year survival rate of less than 10% for those with distant metastases [7,8]. The development of resistance to RAI therapy underscores the need for alternative therapeutic strategies to effectively manage advanced diseases and improve patient outcomes.

The advent of targeted therapies has revolutionized the management of various cancers, including thyroid cancer [9]. Multi-kinase inhibitors (MKIs), which target angiogenesis and tumor proliferation pathways, have emerged as promising therapeutic agents [10,11]. So far, multiple MKIs, have been approved by regulatory agencies and incorporated into treatment guidelines for advanced thyroid cancer [12,13]. However, their use is associated with significant adverse events and high costs, limiting their accessibility and tolerability, particularly in developing countries [14,15].

Surufatinib and donafenib are novel MKIs that have shown efficacy in various solid tumors. Surufatinib targets multiple receptor tyrosine kinases involved in tumor angiogenesis and immune modulation, including VEGFRs, FGFR1, and CSF-1R [16]. Donafenib is a deuterated derivative of sorafenib, designed to enhance molecular stability and pharmacokinetic properties, inhibiting kinases such as VEGFR and PDGFR [17].

Given the limited treatment options for patients with RAIR-DTC and the promising results from initial studies, there is a need to systematically evaluate the efficacy and safety of surufatinib and donafenib in this patient population [18]. This systematic review aims to synthesize current evidence from clinical studies to provide a comprehensive understanding of these agents’ therapeutic potential in DTC.

## 2. Materials and Methods

### 2.1. Eligibility Criteria

The inclusion criteria for this review encompassed studies involving adult patients (≥18 years) diagnosed with differentiated thyroid cancer, specifically focusing on the papillary and follicular subtypes, including cases with locally advanced or metastatic disease. Eligible studies included those where patients were treated with either surufatinib or donafenib and that reported efficacy outcomes such as objective response rate (ORR), progression-free survival (PFS), overall survival (OS), and safety profiles. The review included randomized controlled trials (RCTs), phase II/III clinical trials, and observational studies.

Conversely, studies were excluded if they involved other thyroid cancer subtypes, such as medullary or anaplastic thyroid cancer, unless they provided separate data specific to DTC. Additionally, preclinical studies, abstracts, case reports, reviews, meta-analyses, and studies that lacked sufficient data on predefined outcomes of interest were not included in the analysis.

### 2.2. Information Sources and Search Strategy

For each database, a tailored search strategy was employed using a combination of Medical Subject Headings (MeSH) terms and keywords relevant to the study. Boolean operators (AND, OR) were utilized to refine and optimize the search results. The search was limited to articles published in English until October 2023. Additionally, the references of the retrieved articles were manually screened to identify any studies that might have been missed in the initial search.

PubMed Search Keystring: ((“Surufatinib”[Mesh] OR “Surufatinib”) OR (“Donafenib”[Mesh] OR “Donafenib”)) AND ((“Differentiated Thyroid Neoplasms”[Mesh] OR “differentiated thyroid cancer” OR “thyroid cancer”) OR (“Papillary Thyroid Carcinoma”[Mesh] OR “papillary thyroid cancer”) OR (“Follicular Thyroid Carcinoma”[Mesh] OR “follicular thyroid cancer”)) AND ((“Clinical Trial”[Publication Type] OR “Randomized Controlled Trial”[Publication Type] OR “Phase II Clinical Trial”[Publication Type] OR “Phase III Clinical Trial”[Publication Type] OR “Progression-Free Survival” OR “Overall Survival” OR “Response Rate” OR “Adverse Events”)).

Scopus Search Keystring: (TITLE-ABS-KEY(“Surufatinib”) OR TITLE-ABS-KEY(“Donafenib”)) AND (TITLE-ABS-KEY(“differentiated thyroid cancer”) OR TITLE-ABS-KEY(“thyroid cancer”) OR TITLE-ABS-KEY(“papillary thyroid carcinoma”) OR TITLE-ABS-KEY(“follicular thyroid carcinoma”)) AND (TITLE-ABS-KEY(“clinical trial”) OR TITLE-ABS-KEY(“randomized controlled trial”) OR TITLE-ABS-KEY(“phase II”) OR TITLE-ABS-KEY(“phase III”) OR TITLE-ABS-KEY(“progression-free survival”) OR TITLE-ABS-KEY(“overall survival”) OR TITLE-ABS-KEY(“response rate”) OR TITLE-ABS-KEY(“adverse events”)).

Web of Science Search Keystring: TS = (Surufatinib OR Donafenib) AND TS = (“differentiated thyroid cancer” OR “thyroid cancer” OR “papillary thyroid carcinoma” OR “follicular thyroid carcinoma”) AND TS = (“clinical trial” OR “randomized controlled trial” OR “phase II” OR “phase III” OR “progression-free survival” OR “overall survival” OR “response rate” OR “adverse events”)

Additional search parameters included (1) language restriction; only studies published in English were included to ensure consistency in data extraction and interpretation. (2) Date range: The search was limited to publications available up to October 2023 to capture the most recent and relevant studies. (3) Reference screening: The reference lists of all included articles were manually reviewed to identify any additional studies that met the inclusion criteria but were not captured in the database search.

### 2.3. Selection Process

Data extraction was carried out independently by two reviewers using a standardized data collection form to ensure consistency and accuracy. Extracted data encompassed several key domains: study characteristics (including author, year of publication, study design, sample size, and setting), patient demographics (such as age, sex distribution, disease stage, histological subtype, and prior treatments), and intervention details (covering dosage and administration of surufatinib or donafenib, treatment duration, and any combination therapies used).

For efficacy outcomes, information on objective response rate (ORR), progression-free survival (PFS), overall survival (OS), disease control rate (DCR), time to response (TTR), and duration of response (DoR) were gathered. In terms of safety outcomes, data were extracted on the incidence and severity of adverse events (AEs), treatment-related adverse events (TRAEs), and serious adverse events (SAEs). Any discrepancies between the two reviewers were addressed through discussion or, if needed, by consulting a third reviewer to reach a consensus. The selection process followed the Preferred Reporting Items for Systematic Reviews and Meta-Analyses (PRISMA) protocol [19] to ensure transparency and reproducibility. The registration protocol was registered in the Open Science Framework with the registration code osf.io/gdwcz.

### 2.4. Data Collection and Quality Assessment

The methodological quality of the included studies was assessed using the Cochrane Risk of Bias tool for randomized controlled trials [20]. A qualitative synthesis of the findings was performed due to the heterogeneity of the study designs and outcomes. Descriptive statistics were used to summarize patient demographics, treatment details, efficacy outcomes, and safety profiles. Tables were created to present the key findings systematically. Where available, numerical data, such as hazard ratios (HRs), confidence intervals (CIs), and *p*-values, were reported to indicate the statistical significance of the results.

## 3. Results

**Figure 1:**
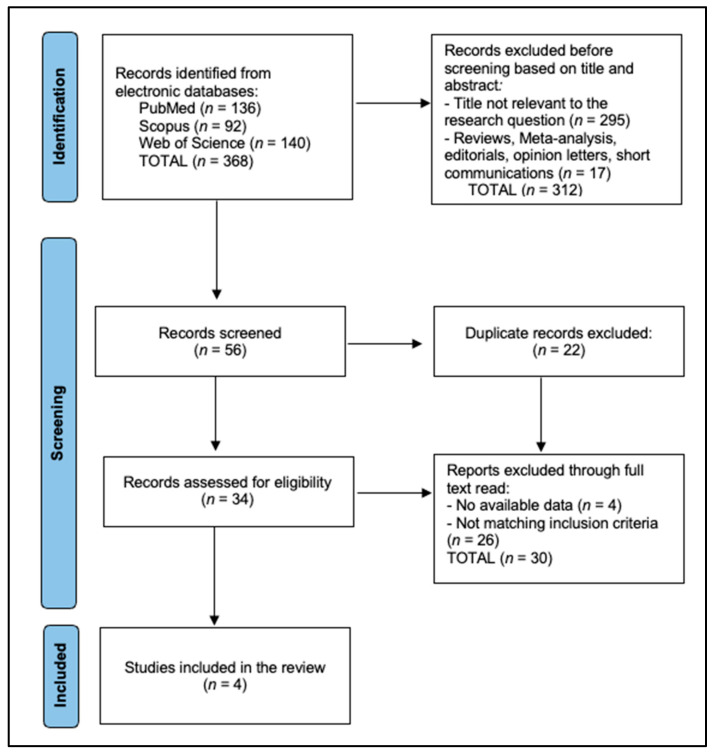
PRISMA flow diagram.

The final analysis included four studies [21,22,23,24], as presented in Figure 1, two on surufatinib and two on donafenib, comprising a total of 291 patients. Chen et al. [21] conducted a phase II trial involving 59 patients with locally advanced or metastatic DTC or medullary thyroid cancer (MTC). Patients received surufatinib 300 mg daily, and the primary endpoint was ORR. The same author, Chen et al. [22], evaluated the efficacy of surufatinib combined with the anti-PD-1 antibody toripalimab in a phase II study with 10 patients having locally advanced DTC. The primary endpoint was ORR in the neoadjuvant setting.

Lin et al. [23] performed a multicenter, randomized, double-blind phase III trial comparing donafenib to placebo in 191 patients with progressive RAIR-DTC. The primary endpoint was PFS. Similarly, Lin et al. [24] conducted a randomized phase II trial comparing two doses of donafenib (200 mg vs. 300 mg twice daily) in 35 patients with locally advanced or metastatic RAIR-DTC, aiming to determine the optimal dosage, as presented in Table 1. All included studies, as summarized in Table 1, were rigorously evaluated using the Cochrane Risk of Bias tool and were determined to be of high quality. This consistent high quality across Phase II and Phase III trials enhances the reliability and credibility of the evidence supporting the efficacy and safety of surufatinib and donafenib in managing advanced differentiated thyroid cancer.

The studies included adult patients ranging from 19 to 79 years of age (Table 2). The majority of patients had an ECOG performance status of 0 or 1, indicating a relatively good functional status. Chen et al. [21] enrolled 59 patients, including both DTC and MTC patients. Most patients had stage IV disease and prior treatments, such as surgery and RAI therapy. In the second study by Chen et al. [22], there were 10 patients with unresectable or borderline resectable locally advanced DTC. The majority were female, and all had measurable disease according to RECIST 1.1.

Lin et al. [23] randomized 191 patients with progressive RAIR-DTC to receive donafenib or placebo. Prior TKI therapy was allowed, with 18.3% of patients having received previous TKI treatment. Moreover, Lin et al. [24] enrolled 35 patients to compare two dosing regimens of donafenib. All patients had RAIR-DTC with measurable disease, and prior TKI therapy was the exclusion criterion (Table 2).

Both surufatinib and donafenib demonstrated efficacy in treating DTC, with notable improvements in ORR and PFS (Table 3). In Chen et al. [21], surufatinib achieved an ORR of 23.2% and a median PFS of 11.1 months. The DCR was high at 87.5%, indicating effective disease control. In addition, Chen et al. [22] reported a higher ORR of 60% with the combination of surufatinib and toripalimab in a neoadjuvant setting. All patients achieved disease control, and 90% underwent successful surgical resection post-treatment.

Lin et al. [23] showed that donafenib significantly improved PFS compared to placebo (12.9 vs. 6.4 months; HR, 0.39; *p* < 0.0001). The ORR was markedly higher in the donafenib group (23.3% vs. 1.7%), and the DCR was also improved. Moreover, Lin et al. [24] compared two dosing regimens of donafenib. Both doses achieved similar ORRs (12.5% vs. 13.3%) and DCRs (100%). The median PFS was longer in the 300 mg arm (15.0 months vs. 9.4 months), suggesting a dose-response relationship.

Both agents exhibited manageable safety profiles, with adverse events consistent with those expected from MKIs (Table 4). Chen et al. [21] reported that the most common grade ≥3 adverse events were hypertension (20.3%) and proteinuria (11.9%). Serious adverse events occurred in 27.1% of the patients, with treatment discontinuation in 13.6%. In addition, Chen et al. [22] reported that most adverse events were grade 1 or 2, with only two patients experiencing grade 3 events (20%). There were no treatment discontinuations due to adverse events.

Lin et al. [23] observed that 99.2% of patients receiving donafenib experienced treatment-related adverse events, with grade ≥3 events in 43.8%. Common severe adverse events included hypertension and hand–foot syndrome. Treatment discontinuation due to adverse events was relatively low at 6.3%. Lin et al. [24] found that both dosing regimens of donafenib were well tolerated. The most common adverse events were palmar–plantar erythrodysesthesia syndrome and hypertension. Grade 3 adverse events occurred in 51.4% of the patients, with no grade 4 or 5 events reported.

It was also observed in a clinical trial by Chen et al. [21] performed in 2020 that the most common grade ≥3 adverse events were hypertension (20.3%) and proteinuria (11.9%). Serious adverse events occurred in 27.1% of patients, with treatment discontinuation in 13.6%. In another study by Chen et al. [22], it was noted that most adverse events were grade 1 or 2, with only two patients experiencing grade 3 events (20%). There were no treatment discontinuations due to adverse events.

Lin et al. [23] observed that 99.2% of patients receiving donafenib experienced treatment-related adverse events, with grade ≥3 events in 43.8%. Common severe adverse events included hypertension and hand–foot syndrome. Treatment discontinuation due to adverse events was relatively low at 6.3%. Moreover, in the other clinical trial results reported by Lin et al. [24] from 2021, it was found that both dosing regimens of donafenib were well tolerated. The most common adverse events were palmar–plantar erythrodysesthesia syndrome and hypertension. Grade 3 adverse events occurred in 51.4% of patients, with no grade 4 or 5 events reported.

## 4. Discussion

### 4.1. Summary of Evidence

The four studies reviewed provide evidence that surufatinib and donafenib are effective treatment options for patients with advanced differentiated thyroid cancer. Both agents demonstrated significant clinical benefits in terms of objective response rates and progression-free survival.

Surufatinib showed promising results in both monotherapy and combination therapy settings. In Chen et al. [21], surufatinib monotherapy achieved an ORR of 23.2% and a median PFS of 11.1 months. The high disease control rate indicates that surufatinib effectively stabilizes disease progression. The combination of surufatinib with toripalimab in Chen et al. [22] led to an even higher ORR of 60%, suggesting potential synergistic effects when combined with immunotherapy. The ability to downstage tumors and facilitate surgical resection highlights its potential in the neoadjuvant setting.

Donafenib demonstrated significant improvements in progression-free survival and objective response rates compared to placebo. In Lin et al. [23], donafenib extended median PFS to 12.9 months, nearly doubling that of the placebo group. The ORR of 23.3% is noteworthy, given the refractory nature of the patient population. The dose-ranging study by Lin et al. [24] suggests that higher doses of donafenib may confer greater benefits in terms of tumor shrinkage and PFS without a proportional increase in severe adverse events.

Both surufatinib and donafenib were generally well tolerated. Common adverse events were consistent with those associated with MKIs, such as hypertension, hand–foot syndrome, and proteinuria. Most adverse events were manageable with dose adjustments or supportive care. Importantly, the rate of treatment discontinuation due to adverse events was low, indicating that patients could maintain therapy to achieve clinical benefits.

The findings from these studies support the incorporation of surufatinib and donafenib into the therapeutic armamentarium for advanced DTC. Their efficacy in improving PFS and ORR offers hope for patients with limited options for RAI-refractory disease. The manageable safety profiles make them suitable for long-term administration, which is critical in the context of chronic cancer management.

Surufatinib and donafenib represent significant advancements in the field of kinase inhibitors, each targeting multiple pathways critical for tumor growth and angiogenesis. Surufatinib, approved in China in late 2020 for the treatment of well-differentiated extrapancreatic neuroendocrine tumors (NETs), selectively inhibits VEGFR 1, 2, and 3, FGFR1, and CSF-1R, offering a targeted approach to managing these tumors [25]. On the other hand, donafenib, also approved in China, in June 2021, is indicated for patients with unresectable hepatocellular carcinoma [26]. This drug is a deuterium derivative of sorafenib, targeting VEGFR, PDGFR, and Raf kinases, providing a valuable option for the first-line treatment of advanced liver cancer. Both approvals underscore the potential of multi-kinase inhibitors to improve therapeutic outcomes across various cancers, including potential applications in thyroid cancer, highlighting a shift toward more personalized and precise cancer therapies.

DONATHYCA is particularly significant given the challenges of radical resection in LATC due to the invasion of critical structures, which increases the risk of recurrence and may even prevent surgery [27]. Although previous studies have shown the survival benefits of donafenib in Chinese patients with radioiodine-refractory differentiated thyroid cancer, its effectiveness in neoadjuvant settings has not been established. The study plans to enroll 13 patients who will receive donafenib 300 mg twice daily over six 21-day treatment cycles. The primary measure of trial success will be the objective response rate, according to the Response Evaluation Criteria in Solid Tumors (RECIST) version 1.1. The secondary outcomes will include progression-free survival, duration of response, disease control rate, R0/R1 resection rate, quality of life, and treatment-related toxicity.

The studies by Marcia S. Brose and colleagues provide critical insights into the timing and efficacy of multi-kinase inhibitors for treating asymptomatic radioactive iodine-refractory differentiated thyroid cancer. In the RIFTOS MKI study [28], the team found that initiating MKI treatment at study entry led to a median time to symptomatic progression (TTSP) of 55.4 months across 647 patients, with those treated immediately (Cohort 1) having a similar TTSP to those whose treatment was deferred (Cohort 2). This large, international, noninterventional study underscored that over 64.5% of patients experienced TTSP of at least 36 months, highlighting the potential prolonged benefit of early MKI intervention. Notably, the median overall survival from classification as RAI-R was an impressive 167 months, with progression-free survival averaging 19.2 months post-MKI therapy initiation. The commonality of treatment-emergent adverse events in 89% of patients, particularly with sorafenib, for which dose modifications were necessary in 70%, reflects the significant management challenges associated with these therapies. In a similar manner, their study [29] also examined the initiation timing of MKIs in a prospective setting, reinforcing the complexity and critical nature of therapeutic timing in asymptomatic RAIR-DTC patients. Both studies highlight the intricate balance between treatment benefits and side effects management in the application of MKIs, advocating for a tailored approach based on individual patient disease progression and overall health status.

Lastly, it is important to acknowledge that MKIs like surufatinib and donafenib have become pivotal in the management of radioactive iodine-refractory differentiated thyroid cancer, offering significant strides in progression-free survival and overall response rates. Despite these advances, the high incidence of toxicities associated with MKIs presents a substantial challenge, often impairing patients’ quality of life [30]. Effective management of RR-DTC with MKIs requires a nuanced approach that includes the assessment of several predictive factors for response and survival. These include clinical markers such as age, performance status, tumor burden, and cancer-related symptoms. Additionally, molecular and biochemical markers, particularly those involved in angiogenic pathways, hold promise for predicting treatment outcomes. The use of MKIs in a real-world Brazilian study highlighted their safety and effectiveness, showing a median progression-free survival of 24 months and an overall survival of 31 months [31]. This study also identified potential prognostic markers, including FDG uptake, maximum SUV of target lesions, and thyroglobulin levels, which correlate with disease progression and patient outcomes. These findings underscore the importance of personalized treatment plans that consider both the therapeutic benefits of MKIs and their potential side effects with the aim of optimizing patient management in RR-DTC.

Su et al. [32] conducted a systematic review and meta-analysis, demonstrating that MKIs significantly improve progression-free survival and overall survival in patients with RR-DTC, with median PFS hazard ratios of 0.30 (95% CI: 0.18–0.50) and OS hazard ratios of 0.70 (95% CI: 0.57–0.88). However, they also noted a higher incidence of adverse events compared to the control groups, urging cautious use based on patient health status to balance treatment benefits against potential harm. Similarly, Hamidi et al. [33] reviewed advancements in treatments for advanced RR-DTC, highlighting the approval and routine use of various targeted therapies, including MKIs. They acknowledged that despite these advances, no current treatments offer a cure, and most patients eventually show disease progression. This emphasizes the ongoing research into resistance mechanisms and novel treatment strategies, suggesting a critical need to enhance the durability of these therapies in clinical practice.

Other MKIs have also been developed recently and tested in clinical trials. In a randomized, double-blind, phase 3 trial, Brose et al. [34] investigated the efficacy of sorafenib in treating patients with radioactive iodine-refractory, locally advanced, or metastatic differentiated thyroid cancer. They found that sorafenib significantly extended median progression-free survival to 10.8 months compared to 5.8 months in the placebo group, with a hazard ratio of 0.59, highlighting its potential as a treatment option despite common adverse effects like hand−foot skin reaction and diarrhea. Similarly, the systematic review by Ligy Thomas and colleagues assessed the broader implications of sorafenib across various types of thyroid cancers, including differentiated and medullary types [35]. They reported a median progression-free survival of 18 months across all subtypes, with significant side effects leading to a high rate of dose reduction or discontinuation. Both studies underscored sorafenib’s role in improving outcomes for patients with advanced thyroid cancer, while also emphasizing the need for meticulous management of its adverse effects to optimize patient tolerance and treatment efficacy.

Another MKI, Lenvatinib, is worth mentioning in this context. Wirth et al. [36] emphasized the importance of early initiation of lenvatinib, particularly before deterioration in Eastern Cooperative Oncology Group performance status, to maximize clinical benefits. They found that a higher initial dose of 24 mg/day, as opposed to 18 mg/day, was associated with better outcomes and a comparable safety profile. Similarly, a randomized study by Brose et al. [37] explored the efficacy and toxicity of starting doses of 18 mg/day versus 24 mg/day of lenvatinib. Their findings did not support the noninferiority of the 18 mg/day dose in terms of objective response rate at week 24, reinforcing the preference for a 24 mg/day starting dose as the more effective regimen for RR-DTC patients, given the marginal difference in high-grade treatment-emergent adverse events between the two doses. Both studies underscored the nuanced approach needed in dosing lenvatinib to balance efficacy and tolerability, advocating tailored treatment strategies based on individual patient profiles and disease burden to optimize outcomes.

Overall, the clinical significance of the improvements in progression-free survival and objective response rate demonstrated by surufatinib and donafenib cannot be understated, particularly in the context of advanced differentiated thyroid cancer, where patients often face limited treatment options. The extension of PFS to 11.1 months with surufatinib and 12.9 months with donafenib, compared to a baseline of 6.4 months with placebo, represents a significant delay in the progression of the disease. This is crucial for patients, as it directly translates to longer periods of stable health without disease worsening, potentially allowing them more normal and active lives. Similarly, the ORR improvements, with surufatinib achieving rates up to 60% and donafenib at 23.3%, indicate that a substantial proportion of patients experience a reduction in tumor size, further contributing to enhanced disease management and quality of life. These metrics collectively highlight the potential of these multi-kinase inhibitors to fill a critical need in the treatment regimen for patients with thyroid cancer who are refractory to conventional therapies, emphasizing their role in extending meaningful clinical benefits to this challenging patient population. The clinical utility of surufatinib and donafenib in the treatment of advanced differentiated thyroid cancer is noteworthy, especially for cases resistant to traditional therapies. These multi-kinase inhibitors offer new options for extending disease control in this patient population with a manageable safety profile. Their potential inclusion in treatment strategies provides significant advancement in the management of resistant cases of thyroid cancer, emphasizing the need for further clinical application and research.

Nevertheless, before initiating any treatment, it is important to understand the pharmacogenetics of these multi-kinase inhibitors. Recent investigations suggest that while no single pharmacogenetic test is yet mandated before initiating surufatinib or donafenib therapy for thyroid cancer, assessing genetic variants in drug-metabolizing enzymes may become an important step toward personalized treatment. In particular, polymorphisms in cytochrome P450 enzymes (especially CYP3A4/5), which are crucial for the metabolism of many tyrosine kinase inhibitors, have been associated with variability in drug clearance and toxicity profiles [38]. Additionally, emerging data indicate that genetic variations in drug transporters such as ABCB1 and metabolic enzymes like UGT1A9 may also influence treatment outcomes and adverse effects [39]. Although further clinical validation is required before these tests can be routinely recommended, incorporating pharmacogenetic screening could eventually help optimize dosing strategies and improve the therapeutic indices of surufatinib and donafenib in oncology.

Emerging evidence indicates that resistance to surufatinib and donafenib in thyroid cancer occurs via multiple interconnected mechanisms. Tumor cells may acquire secondary mutations in the target receptor kinases or activate bypass signaling pathways—such as the fibroblast growth factor (FGF) and platelet-derived growth factor (PDGF) axes—that effectively circumvent the inhibitory actions on VEGFR, FGFR, and CSF1R [40,41]. Additionally, alterations in the tumor microenvironment, including increased levels of hypoxia-inducible factors, can promote the activation of downstream pro-survival cascades, such as the PI3K/AKT and MAPK pathways, further diminishing drug efficacy [42]. These multifaceted resistance mechanisms underscore the potential need for combination therapeutic strategies or multi-targeted approaches to effectively overcome resistance and enhance clinical outcomes in patients thyroid cancer patients.

### 4.2. Limitations

Several limitations of this study should be considered when interpreting these findings. The sample sizes of some studies were relatively small, which may limit the generalizability of the results. These studies were conducted exclusively in Chinese populations, which may not reflect the outcomes in diverse ethnic groups due to genetic and environmental differences. Additionally, long-term overall survival data were not available, and the impact on quality of life was not extensively explored. Direct comparative studies between surufatinib, donafenib, and other approved MKIs are lacking, making it challenging to position these agents fully within existing treatment algorithms.

## 5. Conclusions

Surufatinib and donafenib exhibit significant efficacy in treating advanced differentiated thyroid cancer, providing substantial improvements in progression-free survival and objective response rates with acceptable safety profiles. These agents represent valuable additions to treatment options for patients with RAI-refractory disease. Further large-scale, multicenter studies are warranted to confirm these findings across diverse populations and to establish their roles in combination therapies and in comparison with other standard treatments.

## Figures and Tables

**Table 1:** Summary of the included studies.

Study and Author	Year	Country	Study Design	Sample Size	Study Population	Study Quality
Chen et al. [21]	2021	China	Phase II, Open-Label Trial	59	Locally Advanced/ Metastatic DTC	High
Chen et al. [22]	2023	China	Phase II, Single-Arm Study	10	Locally Advanced DTC	High
Lin et al. [23]	2023	China	Phase III, Randomized Controlled	191	Progressive RAIR-DTC	High
Lin et al. [24]	2020	China	Phase II, Randomized Trial	35	Locally Advanced/Metastatic RAIR-DTC	High

DTC—Differentiated Thyroid Cancer; RAIR—Radioactive Iodine-Refractory.

**Table 2:** Patient demographics and baseline characteristics.

Study and Author	Mean Age (Years)	Sex (Male/Female)	Histological Subtype	Prior Treatments
Chen et al. [21]	19–78 (Median 59)	28/31	DTC and MTC	Surgery, RAI, Chemotherapy
Chen et al. [22]	28–76 (Median 59)	2/8	Locally Advanced DTC	Surgery, RAI
Lin et al. [23]	27–76 (Median 59)	84/107	RAIR-DTC	Surgery, RAI, TKI Therapy
Lin et al. [24]	21–79 (Median 55)	13/22	RAIR-DTC	Surgery, RAI

DTC—Differentiated Thyroid Cancer; MTC—Medullary Thyroid Cancer; RAIR-DTC—Radioactive Iodine-Refractory Differentiated Thyroid Cancer; RAI—Radioactive Iodine; TKI—Tyrosine Kinase Inhibitor.

**Table 3:** Treatment details and efficacy outcomes.

Study and Author	Treatment Regimen	ORR (%)	Median PFS (Months)	DCR (%)
Chen et al. [21]	Surufatinib 300 mg daily	23.2	11.1	87.5
Chen et al. [22]	Surufatinib + Toripalimab	60	Not Reported	100
Lin et al. [23]	Donafenib 300 mg twice daily	23.3 (vs. 1.7 placebo)	12.9 (vs. 6.4 placebo)	93.3 (vs. 79.3 placebo)
Lin et al. [24]	Donafenib 200 mg vs. 300 mg twice daily	12.5 vs. 13.3	9.4 vs. 15.0	100 in both arms

ORR—Objective Response Rate; PFS—Progression-Free Survival; DCR—Disease Control Rate.

**Table 4:** Safety profiles and adverse events.

Study and Author	Common Adverse Events	Grade ≥ 3 AEs (%)	Treatment Discontinuation Due to AEs (%)
Chen et al. [21]	Hypertension, Proteinuria, PPE	43.8	13.6
Chen et al. [22]	Hypertension, Bilirubin Increase	20	0
Lin et al. [23]	HFS, Hypertension, Diarrhea	43.8	6.3
Lin et al. [24]	PPE, Hypertension, Alopecia	51.4	Not Reported

AE—Adverse Event.

## Data Availability

Not applicable.
